# TMEM176B inhibits ovarian cancer progression by regulating EMT via the Wnt/β-catenin signaling pathway

**DOI:** 10.1186/s12967-025-06362-0

**Published:** 2025-03-19

**Authors:** Lili Yan, Zhaona Song, Lili Yi, Conghui Tian, Ruirui Zhang, Xuying Qin, Xiang Wang, Shaoda Ren, Xiaoping Ma, Xiaobing Wang, Xiaofeng Zhao, Feifei Wang, Jianmei Wei, Xiaodong Jia, Mingliang Gu, Fengjiao Yuan, Dianlong Jia

**Affiliations:** 1 Joint Laboratory for Translational Medicine Research, Liaocheng People’s Hospital, Liaocheng, Shandong 252000 PR China; 2 State Key Laboratory of Macromolecular Drugs and Large-scale Preparation, School of Pharmaceutical Sciences and Food Engineering, Liaocheng University, Liaocheng, Shandong 252059 PR China; 3 Department of Pathology, Liaocheng People’s Hospital, Liaocheng, Shandong 252000 PR China; 4 Department of Obstetrics and Gynecology, Liaocheng People’s Hospital, Liaocheng, Shandong 252000 PR China; 5 Central Laboratory of Liaocheng People’s Hospital, Liaocheng, Shandong 252000 PR China; 6 Shandong Provincial Key Medical and Health Discipline of Liaocheng Tumor Hospital, Liaocheng, 252000 PR China

**Keywords:** Ovarian cancer, TMEM176B, Epithelial-to-mesenchymal transition, Wnt/β-catenin signaling pathway

## Abstract

**Background:**

Ovarian cancer (OC) is recognized as one of the deadliest forms of gynecological cancer, approximately two-thirds of patients have already developed metastasis when they are diagnosed. The function of transmembrane protein 176B (TMEM176B) in the progression of OC remains elusive. This study aimed to investigate the role and molecular mechanism of TMEM176B on OC proliferation and metastasis.

**Method:**

Expression of TMEM176B in OC and normal tissues were determined from the TCGA, GTEx, and CPTAC databases, and verified by patient-derived tissue samples. We analysed the prognostic relevance of TMEM176B in OC via Kaplan‒Meier (K‒M) survival curves and receiver operating characteristic (ROC) curves. Subsequent in vitro assays, including the CCK8 assay, colony formation assay, wound healing assay, and transwell assay, were performed to detect the influence of TMEM176B on cell proliferation and metastasis. Furthermore, a tumorigenesis study in nude mice was conducted to confirm the suppressive impact of TMEM176B on OC. RNA sequencing (RNA-seq) was utilized to uncover the mechanisms of TMEM176B on OC progression. Spearman correlation analysis was used to calculate the correlations between TMEM176B and cell adhesion, DNA replication, and the Wnt/β-catenin pathway. Finally, the role of TMEM176B in regulating the epithelial-mesenchymal transition (EMT) depending on the Wnt/β-catenin pathway was evaluated using LiCl agonist.

**Result:**

The mRNA expression of TMEM176B was significantly downregulated in OC tissues, with lower TMEM176B correlating with a worse prognosis. Moreover, higher tumor stage and tumor grade were associated with a lower TMEM176B protein level. Consistent with these findings, OC tissues exhibited significantly reduced of TMEM176B compared to normal ovarian tissue from patients. In vitro studies indicated that TMEM176B knockdown increased both the proliferation, metastasis and EMT levels of OC cells, while TMEM176B overexpression had the opposite effects. In vivo investigations reinforced that TMEM176B significantly inhibited the progression of OC. RNA-seq analysis demonstrated that TMEM176B enhanced cell adhesion, diminished DNA replication, and suppressed EMT through the regulation of the Wnt/β-catenin signaling pathway, effectively obstructing the proliferation and metastasis of OC cells and impeding the disease’s progression.

**Conclusions:**

TMEM176B inhibited EMT in OC cells by controlling the activation of the Wnt/β-catenin pathway. This mechanism underscored the diagnostic and prognostic potential of TMEM176B for OC and highlights its tumor-suppressive properties as a promising therapeutic candidate.

**Supplementary Information:**

The online version contains supplementary material available at 10.1186/s12967-025-06362-0.

## Introduction

Ovarian cancer (OC) represents a malignant neoplasm impacting the female reproductive system, characterized by a notably high incidence rate, making it one of the most lethal cancers globally [1]. Despite substantial advancements in cancer research, a majority of OC patients receive a diagnosis at an advanced stage, often associated with a bleak prognosis and limited treatment alternatives primarily due to a lack of effective screening methods [2]. Consequently, there has been no significant enhancement in overall survival for these individuals over recent decades. Although recent research has uncovered considerable information regarding the onset and origin of OC tumors [3], a key challenge in achieving effective treatments is the insufficient grasp of the biological mechanisms governing tumor dissemination and metastasis [4]. Thus, a thorough understanding of the processes behind OC development and metastasis is essential.

The transmembrane protein (TMEM) family, which comprises integral components of cellular membranes, plays vital roles in biological development, differentiation, immune responses, and the progression of cancer [5]. TMEM176B, known as tolerance-related and induced transcript (TORlD), is a tetraspanin membrane protein recognized for its widespread expression, comprising four transmembrane domains along with an immunoreceptor tyrosine-based inhibitory motif (ITIM) located at its C-terminus [6]. Phylogenetic studies indicate that TMEM176B originated in cartilaginous fish, where it was initially expressed in non-immune cells before being adopted by immune cells across mammals [7]. Presently, TMEM176B is acknowledged as an immunomodulatory cation channel, emphasizing its role in regulating immune cell functions [8, 9]. This protein inhibits maturation and enhances MHC class II antigen presentation to naive CD4^+^ T cells within dendritic cells [10, 11]. Moreover, TMEM176B plays a role in the immune response against SARS-CoV-2 as well as in antitumor immunity by modulating inflammasome activation [12, 13]. Notably, a growing body of research supports the notion that TMEM176B might be instrumental in tumor initiation and progression. For instance, inhibitors of TMEM176B can enhance the antitumor efficacy of CD146^+^ tumor-associated macrophages [14]. TMEM176B is known to significantly promote the progression of gastric cancer [15], whereas it has a favorable prognosis for melanoma patients [16]. Consequently, TMEM176B is regarded as a valuable diagnostic and prognostic biomarker for various malignant tumors.

Metastasis of tumors is the leading cause of death among OC patients [17]. Throughout the metastatic process, cells undergo epithelial-mesenchymal transition (EMT). This transition from an epithelial phenotype to a mesenchymal phenotype enables cells to detach from their originating tissue and disseminate throughout the organism, thereby enhancing the migratory and invasive capacity of tumor cells [18, 19]. During EMT, numerous molecular, morphological, and functional alterations transpire, including changes in cell-matrix adhesion, cellular polarity, intercellular signaling, and cytoskeletal rearrangements [20]. In summary, EMT functions as an escape strategy within cancer cells, and its reactivation is critical to cancer progression [21]. A recent investigation demonstrated that overexpression of TMEM176B promotes EMT through the FGFR/JNK signaling pathway in lung adenocarcinoma [22]. However, the roles and mechanisms of TMEM176B concerning the development, progression, and EMT of OC have yet to be elucidated.

In our research, we established the expression pattern of TMEM176B in OC and demonstrated its significant correlation with clinical factors. Our objective was to gain a deeper insight into the role and molecular mechanism involving TMEM176B in the proliferation and metastasis of OC cells. We expect identifying novel therapeutic targets and improved treatment strategies for OC.

## Materials and methods

### Reagents

Cell Counting Kit-8 (CCK-8) was purchased from MedChem Express (Shanghai, China). c-Myc (18583 S), Lamin B1 (13435 S), β-Actin (3700 S) and FLAG (14793 S) antibodies were acquired from Cell Signaling Technology (MA, USA). E-cadherin (20874-1-AP), MMP9 (10375-2-AP), β-catenin (51067-2-AP), SNAI2 (12129-1-AP), Cyclin D1 (60186-1-Ig), TCF7 (14464-1-AP), and LEF1 (14972-1-AP) antibodies were obtained from Proteintech (IL, USA). TMEM176B (A24509) antibody was obtained from ABclonal (Wuhan, China). qPCR-related reagents were purchased from Vazyme (Nanjing, China). Matrigel matrix glue was purchased from BD Biosciences (New Jersey, USA). Western blotting-related reagents were purchased from Beyotime (Nanjing, China). The agonist of Wnt/β-catenin (LiCl, HY-Y0649) was purchased from MedChem Express (Shanghai, China). Stock solutions of the drug was made with dimethyl sulfoxide (DMSO; Sigma‒Aldrich, USA) and diluted in Dulbecco’s modified Eagle medium (DMEM; Gibco, USA) to final concentrations for the cell experiments. The final concentration of DMSO was ≤ 0.1%.

### Tissue samples

Three pairs of tissue from OC patients were analysed, which contained normal and cancerous ovarian tissues with definite pathological diagnoses. The criteria for tissue included an original histological diagnosis of OC, and the efficiency of clinical pathological data. The study was conducted in accordance with the Declaration of Helsinki and was approved by The Ethical Committee of Liaocheng People’s Hospital. Each patient provided informed consent.

### Immunohistochemistry (IHC) assay

The expression of TMEM176B protein was verified by IHC. The study included patients diagnosed with OC who were admitted to Liaocheng People’s hospital between January to December 2024. Paraffin-embedded ovarian tissue were collected from these patients. A total of 5 normal ovarian tissues, 5 primary OC tissues, and 5 metastatic OC tissues were gathered and analysed using anti-TMEM176B antibody. The IHC procedure was performed as described as the manufacturer’s instructions of the kit (KIT-9706, MXB Biotechnologies, Fuzhou, China).

### Cell culture and generation of stably transfected cell lines

The human OC cell lines A2780, SKOV3, and OVCAR3, were cultured in DMEM supplemented with 10% fetal bovine serum (FBS; Gibco, USA) at 37°C in a 5% CO_2_ atmosphere. A2780 cells were infected with the negative control (LVNC) or TMEM176B overexpression (LVTMEM176B) lentivirus. The human TMEM176B (NM_001362691) sequence was synthesized with the following primers: forward: 5’-AGGTCGACTCTAGAGATCCCGCCACCATGACAGCACAGAACTTGG‐3’ and reverse: 5’‐TCCTTGTAGTCATGGATCCCAGGACAACAATGGCAGTGGAGGTTG‐3’ and packaged on the lentivirus vector plasmid GV703 (CMV enhancer-MCS-3FLAG-EF1a-ZsGreen1-T2A-puromycin) by GeneChem (Shanghai, China). The control shRNA (shNC) and TMEM176B shRNA (shTMEM176B) lentivirus purchased from Santa Cruz Biotechnology (sc-89659-V, California, USA). OVCAR3 cells were infected with the shNC or shTMEM176B lentivirus. After 48 h of transfection, cells were selected with 2 µg/mL puromycin for 48 h. Total RNA and protein from the transfected cells were extracted, and analysed via RT-qPCR or western blotting to confirm the expression of TMEM176B in stable cells.

### RNA extraction and real-time quantitative PCR

Total RNA was extracted from the cells using TRIzol reagent based on the manufacturer’s guidelines, then resuspended in RNase-free water and quantified with a NanoDrop spectrophotometer (Thermo Fisher Scientific, USA). cDNA was transcribed using the HiScript^®^ III RT SuperMix for qPCR Kit, and quantitative real-time polymerase chain reaction (qRT-PCR) was conducted utilizing the ChamQ SYBR qPCR Master Kit. The relative expression levels of mRNA were assessed with an ABI QuantStudio Q5 analyzer (Applied Biosystems, Thermo Fisher, USA) and analysed using the 2^−ΔΔCt^ method, with β-Actin serving as the internal control. The upstream primer for human β-Actin was 5’-CCTCGCCTTTGCCGATCC-3’, and the downstream primer was 5’-GGATCTTCATGAGGTAGTCAGTC-3’. The upstream primer for human TMEM176B was 5’-CCCTACCACTGGGTACAGATGGA-3’, and the downstream primer was 5’-CTTCAAGACACAGACAGCCAGGA-3’.

### Western blotting

The cells from each group were harvested. Proteins were separated by sodium dodecyl sulfate-polyacrylamide gel electrophoresis (SDS‒PAGE) and transferred onto polyvinylidene difluoride (PVDF) membranes. The membranes were blocked with a low background rapid blocking solution (Kermey Biotech, China) for 20 min and then incubated with primary antibodies overnight at 4 °C. The membranes were subsequently incubated with secondary antibodies for 1 h at room temperature. Finally, the proteins were detected via a Gel Doc Imaging System (ChemiDoc MP, Bio-Rad, USA), and the gray values were evaluated with ImageJ software (National Institutes of Health, USA).

### Cell proliferation and colony formation

For the cell proliferation assays, OVCAR3 and A2780 cells in the logarithmic growth phase were seeded in 96-well plates (Corning Costar, USA) at a density of 4,000 cells per well. Proliferation was measured via the CCK8 assay as described in the manufacturer’s manual at days 1, 2, 3, 4, and 5. For the colony formation assays, the cells were seeded in 12-well culture plates at a density of 350 cells per well and cultured for 7 to 10 days. Culturing was stopped when the number of single-cell colonies was greater than or equal to 50. The colonies were then fixed and stained with 100% methanol and a dye solution containing 0.1% crystal violet, respectively. The number of colonies was counted and analysed.

### Wound healing

For wound healing assays, OVCAR3 and A2780 cells in the logarithmic growth phase were seeded in 6-well plates and incubated until they reached confluence. The monolayer of cells was scratched in a straight line via a sterile pipette tip and washed with PBS to remove detached cells. The 6-well plates were then placed in an incubator for continued culture. Images were taken at the same position at 0 h and 48 h. The scratch area was measured via ImageJ software.

### Transwell migration and invasion assay

Cell migration and invasion assays were performed in 24-well plates (Corning Costar, USA). For the migration assay, 200 µl of serum-free growth medium containing 40,000 cells was added to the upper chamber, while 600 µl of medium containing 20% FBS, which acts as a chemoattractant, was added to the lower chamber simultaneously. For the invasion assay, Matrigel matrix gel was diluted 1:5 with serum-free medium, and 50 µl of the diluted gel was added to the upper chamber and placed in the incubator for 4 h. Then, 200 µl of serum-free growth medium containing 50,000 cells was added to the upper chamber, whereas 600 µl of medium containing 20% FBS, which acts as a chemoattractant, was added to the lower chamber simultaneously. After 24 h of incubation, the migrated cells were stained with 100% methanol and a dye solution containing 0.1% crystal violet, followed by imaging and counting under an inverted microscope (Nikon, Tokyo, Japan).

### In vivo assays

Female BALB/c nude mice (4–6 weeks old) were purchased from Jinan Pengyue Laboratory Animal Breeding. For the tumorigenesis assay, 1 × 10^7^ A2780 cells overexpression TMEM176B and negative control were resuspended in 200 µl of sterile PBS and injected subcutaneously into randomly selected mice (*n* = 5 per group). To track the process of tumor formation, the volume of xenografts and the weight of the mice were measured every 2 days after the appearance of the tumors. When palpable tumors reached 1.0 cm in diameter, they were surgically excised and weighed. This study was conducted in accordance with the principles of the Declaration of Helsinki. Animal experiments were conducted according to the guidelines of the Institutional Animal Care and Use Committee of the Model Animal Research Center, and were approved by the Ethics Committee of Liaocheng People’s Hospital.

### RNA sequencing and data analysis

Total RNA from A2780 cells was extracted via a TRIzol assay kit. After purification of the total RNA, RNA integrity was evaluated via the Agilent Fragment Analyser 5400 system. RNA library construction, sequencing, and data processing were carried out as previously described [23]. Gene Ontology (GO) and Kyoto Encyclopedia of Genes and Genomes (KEGG) pathway enrichment analyses were performed via the R package “clusterProfiler”. Gene set enrichment analysis (GSEA) was conducted via the R package “clusterProfiler”, and the results were presented through the package “enrichplot”. The above experiment was completed with the assistance of Novogene (Beijing, China).

### Gene expression profiling analysis

The gene expression profiles and clinical data of TCGA-OC patients (*n* = 376) were downloaded from the UCSC Xena (https://xenabrowser.net/datapages/) database. We also obtained the transcriptome profiles of normal tissues as controls (*n* = 180) from the Genotype-Tissue Expression database (GTEx, https://gtexportal.org/). The batch effects among the two different datasets (TCGA and GTEx) were corrected through the “Combat” algorithm via the “SVA” package of R software. Based on the criteria of |log2FoldChange| > 0 and *P* value < 0.05, the differentially-expressed genes (DEGs) between TCGA-OC and normal tissues were identified. TCGA-OC samples were divided into two groups according to the median expression of TMEM176B, and prognostic differences between different groups were assessed via Kaplan‒Meier (K‒M) survival curves. We analysed the proteomic expression profiles of TMEM176B in OC and normal tissues based on clinical factors, including sample types, tumor stages, tumor grades, ages, and Wnt pathway status, in the Clinical Proteomic Tumor Analysis Consortium (CPTAC) database (https://ualcan.path.uab.edu/analysis-prot.html). Spearman correlation analysis was applied to calculate the correlation coefficients. The STRING database (https://cn.string-db.org/) was used to identify the protein-protein interaction network.

### Statistical analysis

All the bioinformatic statistical analyses were conducted with R software (version 4.2.2). For RNA-seq analysis, the statistical analysis is described in the “RNA sequencing and data analyses” section. The significance of the data between two experimental groups was determined via Student’s *t*-test, and multiple group comparisons were analysed via one-way ANOVA. Statistical analyses were performed via GraphPad Prism 9.0 software. *P < 0.05, **P < 0.01, and ***P < 0.001 indicate significance.

## Results

### TMEM176B was significantly decreased in OC and associated with poor prognosis

**Fig. 1:**
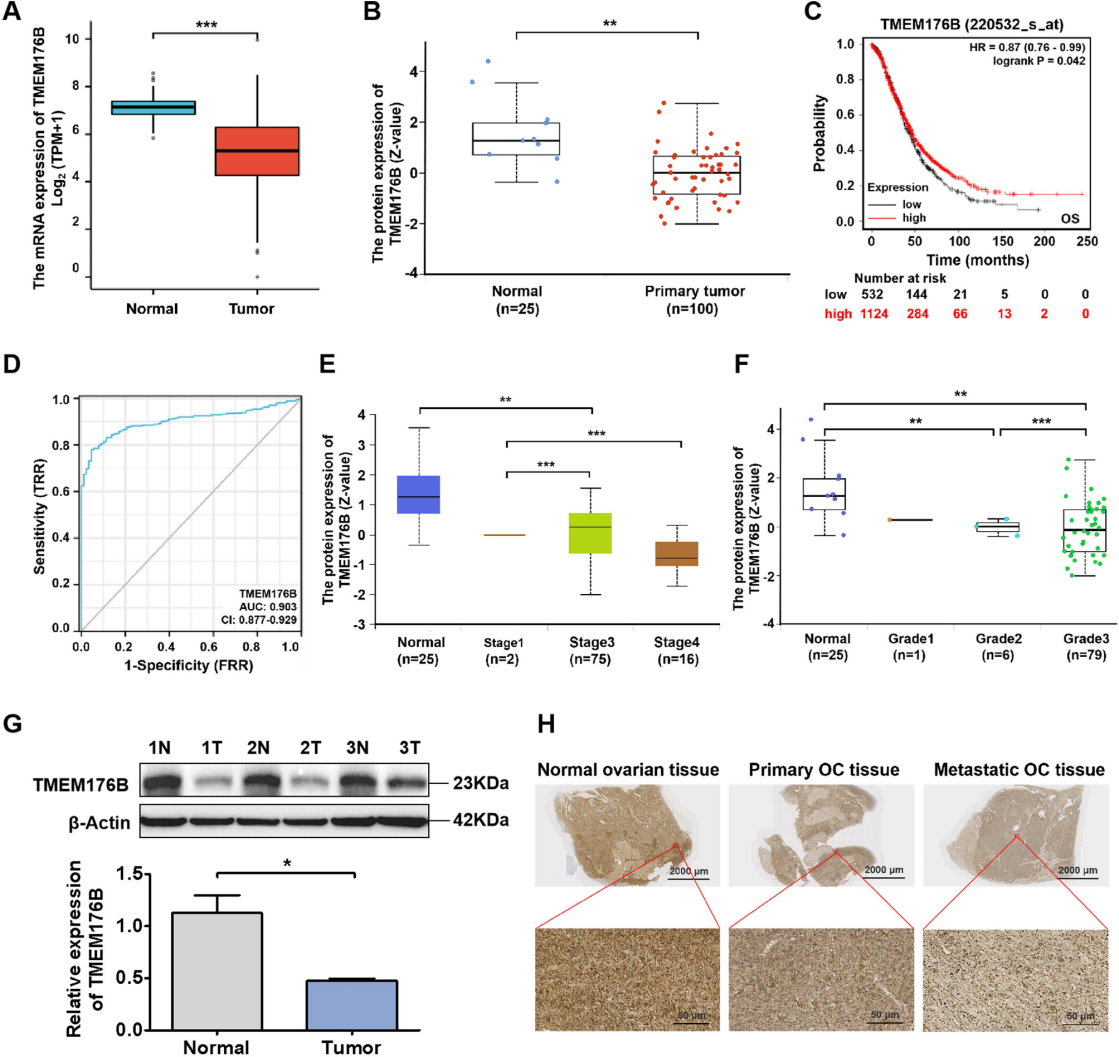
TMEM176B expression was significantly decreased in OC and was associated with prognosis. (**A**) The mRNA expression level of TMEM176B in OC (*n* = 376) was significantly decreased compared to normal tissue (*n* = 180) based on TCGA and GTEx datasets. (**B**) The protein expression level of TMEM176B in OC was significantly decreased compared to normal tissue based on the CPTAC database. (**C**) Patients with lower TMEM176B expression was associated with worse OS. (**D**) ROC analysis showed that TMEM176B could identify tumor and normal tissue, and the AUC was 0.903. The protein expression level of TMEM176B was significantly decreased in different tumor stages (**E**) and tumor grades (**F**) of OC patients based on the CPTAC database. (**G**) The protein expression of TMEM176B in tumor tissue (Tumor; T) and contralateral normal ovarian tissue (Normal; N) from three OC patients was detected by Western blotting. (**H**) The expression of TMEM17B in the ovarian tissue from healthy individuals, primary OC patients, and OC patients with metastasis was analysed using immunohistochemistry. Scale bars, 2000 μm and 50 μm. *P < 0.05, *P < 0.05; **P < 0.01; ***P < 0.001

To investigate the potential role of TMEM176B in OC development, we detected the expression of TMEM176B in paired OC samples based on TCGA, GTEx, and CPTAC datasets. TMEM176B mRNA expression was significantly lower in OC tumor tissues compared to matched normal tissues (Fig. 1A). Similarly, analysis of the CPTAC database revealed a consistent reduction in TMEM176B protein levels in OC tumor tissues compared to normal tissues (Fig. 1B). The OC patients were divided into high- and low-expression groups based on the median expression level of TMEM176B, and the impact of TMEM176B on the prognosis of OC patients was evaluated. Moreover, our results indicated that patients with lower TMEM176B expression had a worse overall survival (OS) (Fig. 1C). The area under the curve (AUC) derived from the ROC curve was 0.903 (Fig. 1D). Furthermore, according to the CPTAC database, significant differences in TMEM176B protein expression were observed across various tumor stages (Fig. 1E), tumor grades (Fig. 1F), and patient age groups (Fig. S1). Higher tumor stage and grade were associated with a lower TMEM176B protein level. Consistent with these findings, OC tissues exhibited significantly reduced protein and mRNA levels of TMEM176B compared to contralateral normal ovarian tissue from OC patients (Fig. 1G and Fig. S2). IHC analysis further revealed that the expression of TMEM176B in primary OC was markedly lower than that in normal ovarian tissue, and the expression was even lower after tumor metastasis (Fig. 1H). These results suggest that TMEM176B plays an important role in OC progression and could be used as a diagnostic and prognostic indicator of OC.

### TMEM176B knockdown promoted OC cell proliferation and metastasis in vitro

**Fig. 2:**
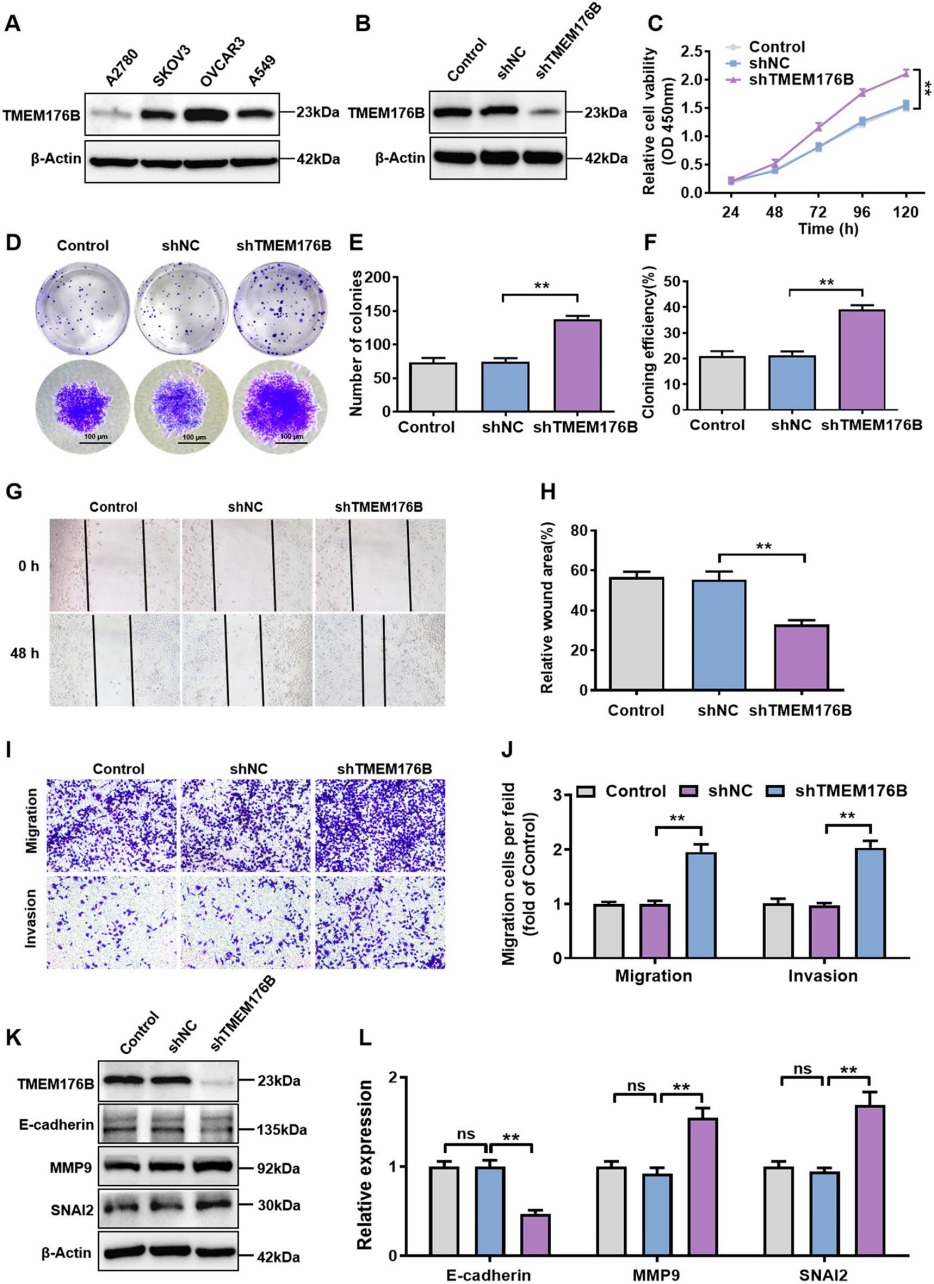
TMEM176B knockdown promoted OC cells proliferation and metastasis in vitro. (**A**) The protein expression levels of TMEM176B in various OC cell lines, with A549 as a positive control. (**B**) OVCAR3 cells were transfected with shNC or shTMEM176B lentiviruses. The knockdown efficiency was verified by Western blotting. (**C**) The effect of TMEM176B knockdown on the proliferation of OVCAR3 cell line. (**D-F**) The effect of TMEM176B knockdown on the colony-forming ability of OVCAR3 cells. (**G-H**) Wound healing assays were used to evaluate the ability of TMEM176B knockdown on cell migration. The histogram showed the statistical analysis of the wound healing rate at 48 h. Scale bars, 500 μm. (**I-J**) The effect of TMEM176B knockdown on the migration and invasion of OVCAR3 cells was assessed by transwell assay. Scale bars, 100 μm. (**K-L**) The effect of TMEM176B knockdown on the protein levels of EMT markers. **P < 0.01, *n* = 3

The frequent silencing of TMEM176B in OC tissues (Fig. 1) suggested that this gene may be a tumor suppressor. To investigate the biological roles of TMEM176B in OC, we detected TMEM176B protein levels in various OC cell lines and found that TMEM176B was highly expressed in OVCAR3 cells but lowly expressed in A2780 cells (Fig. 2A). Therefore, we stably knocked down TMEM176B in OVCAR3 cells by transfecting shRNA lentivirus (Fig. 2B and Fig. S3). The CCK-8 assays showed that the cell proliferation ability of the TMEM176B knockdown group was significantly enhanced (Fig. 2C). Meanwhile, The colony formation assays revealed that TMEM176B knockdown not only augmented the colony size but also increased the colony number and formation rate (Fig. 2D-F). Furthermore, wound healing and transwell assays were conducted to elucidate the effect of TMEM176B silencing on the metastasis of OC cells. Compared with the control groups, TMEM176B knockdown significantly promoted the metastasis of OVCAR3 cells in vitro (Fig. 2G-J). Additionally, the knockdown of TMEM176B significantly increased the expression of the EMT markers MMP9 and SNAI2 while decreased the expression of E-cadherin (Fig. 2K-L).

### TMEM176B overexpression inhibited OC cell proliferation and metastasis in vitro

**Fig. 3:**
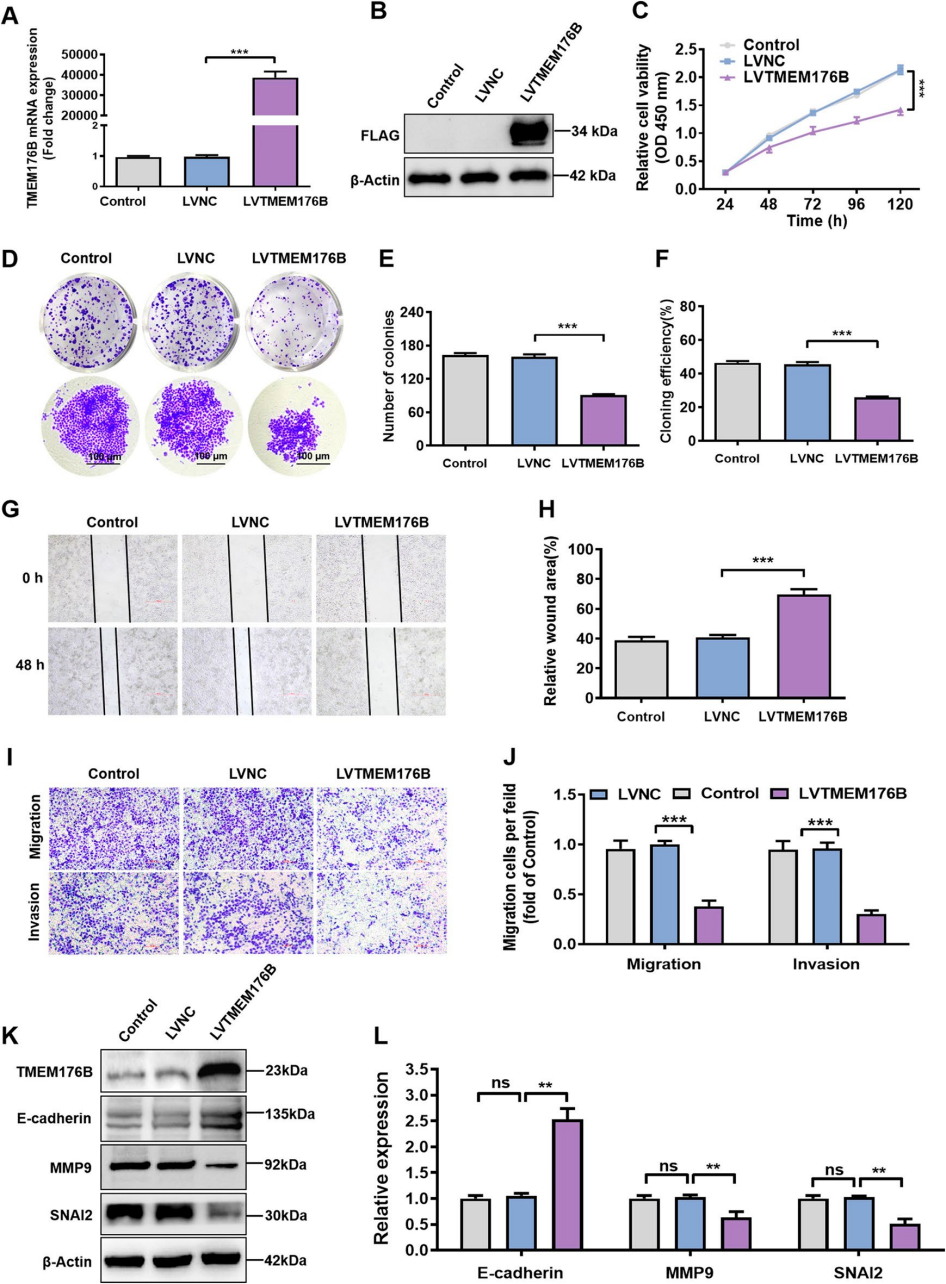
TMEM176B overexpression inhibited OC cells proliferation and metastasis in vitro. A2780 cells were transfected with negative control or TMEM176B overexpression lentiviruses. qPCR (**A**) and Western blotting (**B**) was used to determine the efficiency of TMEM176B overexpression in A2780 cell line. (**C**) The effect of TMEM176B overexpression on the proliferation of A2780 cell line. (**D-F**) The effect of TMEM176B overexpression on the colony-forming ability of A2780 cells. (**G-H**) Wound healing assays was used to evaluate the ability of TMEM176B overexpression on cell migration. The histogram showed the statistical analysis of the wound healing rate at 48 h. Scale bars, 500 μm. (**I-J**) The effect of TMEM176B overexpression on the migration and invasion of A2780 cells was assessed by transwell assay. Scale bars, 100 μm. (**K-L**) The effect of TMEM176B overexpression on the protein levels of EMT markers. **P < 0.01; ***P < 0.001, *n* = 3

To further confirm the inhibitory effect of TMEM176B on OC, we also constructed A2780 cell line with stable overexpression of TMEM176B (Fig. 3A-B, and Fig. S3). On the contrary, overexpression of TMEM176B inhibited the ability of cell proliferation (Fig. 3C), colony formation (Fig. 3D-F), and migration (Fig. 3G-J) of A2780 cells. Furthermore, TMEM176B overexpression decreased the expression of MMP9 and SNAI2 but increased the expression of E-cadherin (Fig. 3K-L). These results suggest that TMEM176B is a negative regulator that inhibits the proliferation and metastasis of OC in vitro.

### TMEM176B suppressed the growth of OC xenografts in vivo

**Fig. 4:**
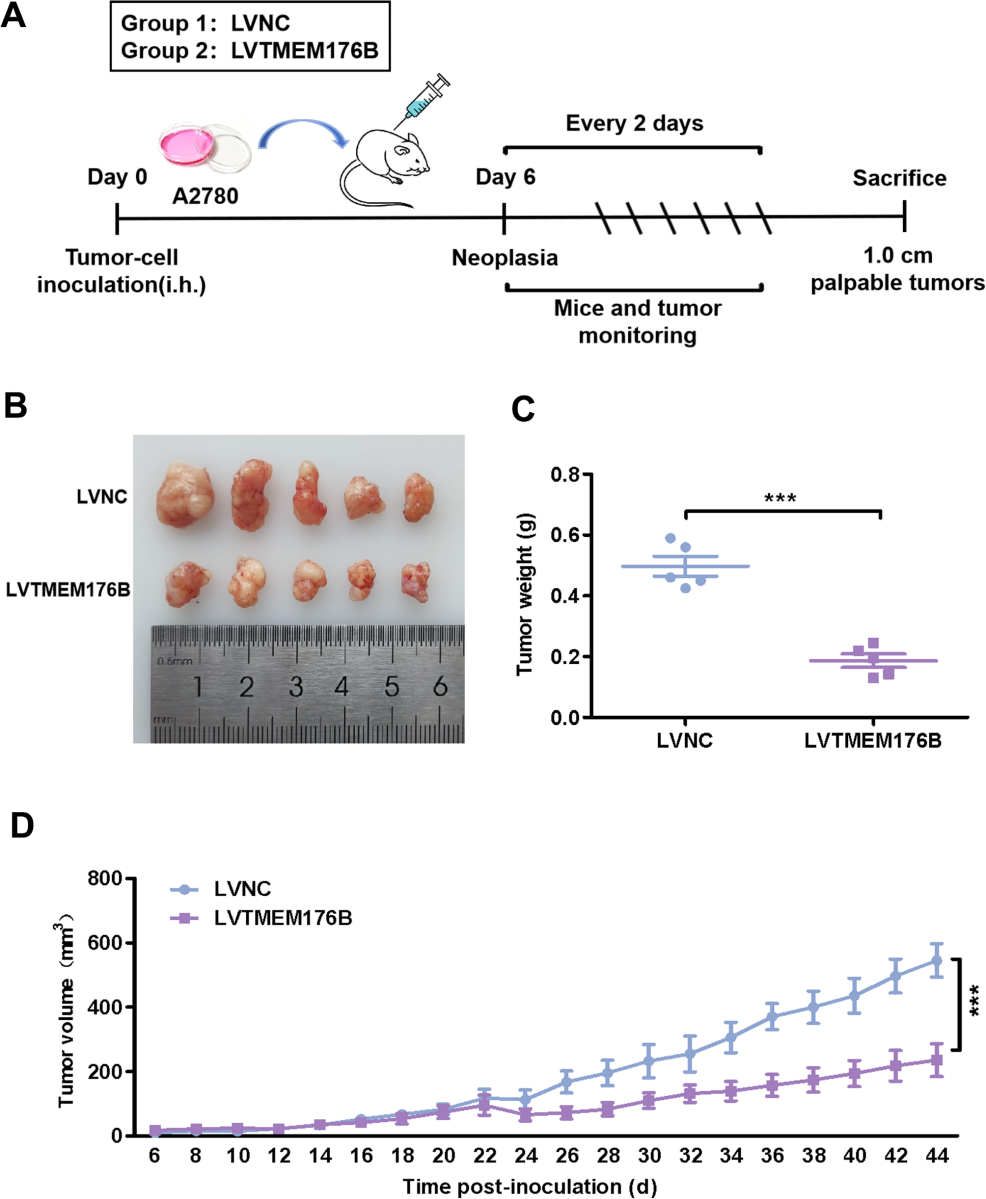
TMEM176B suppressed tumor growth of OC cells in vivo. A2780 cells were transfected with negative control or TMEM176B overexpression lentivirus, and then injected subcutaneously into mice. (**A**) Schematic representation of the subcutaneous heterograft tumor model in nude mice. (**B-C**) Changes in tumor size and weight in the model mice. (**D**) Changes in tumor volume in the model mice. ***P < 0.001, *n* = 5

We further assessed the role of TMEM176B in OC tumorigenesis in vivo by establishing a subcutaneous xenograft model using A2780 cells in female BALB/c nude mice. As shown in the schematic representation, A2780 cells with TMEM176B overexpression or negative control cells were injected subcutaneously into the mice (*n* = 5) (Fig. 4A). Once the tumors formed, tumor growth was measured every other day. Our results revealed that, compared with the negative control, the overexpression of TMEM176B in A2780 cells led to a significant decrease in tumor mass and volume (Fig. 4B-C). Moreover, tumors in the TMEM176B overexpression group began to grow more slowly from the 24th day onwards (Fig. 4D). During the monitoring of tumor growth, the weights of the mice in both groups were not adversely affected (Fig. S4). Together, these findings indicate that TMEM176B prevents the progression of OC in vivo.

### TMEM176B regulated cell adhesion and DNA replication

**Fig. 5:**
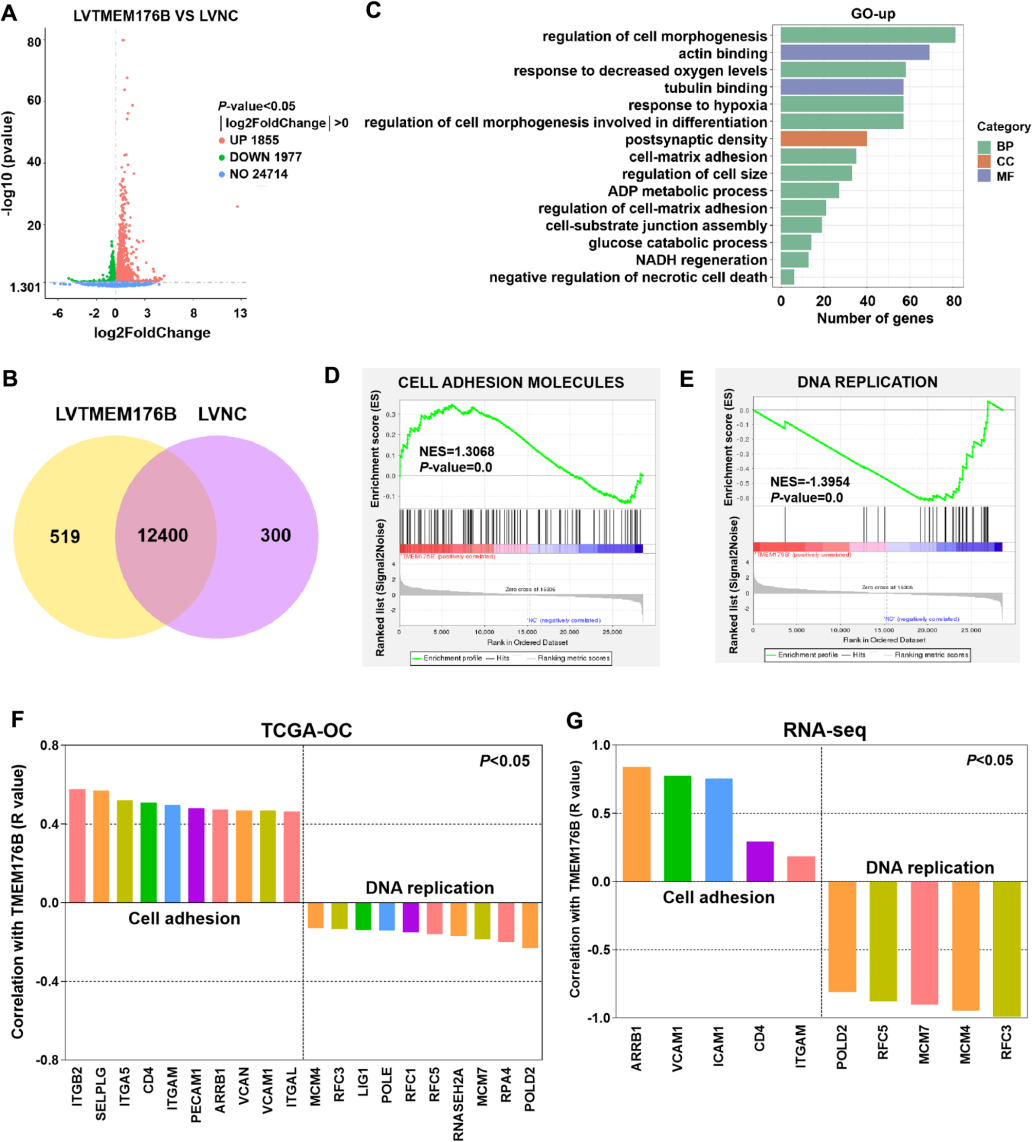
TMEM176B regulated the cell adhesion and DNA replication. RNA-seq analysis was performed on TMEM176B overexpression and negative control A2780 cells, *n* = 3. (**A**) The volcano plot showed the up and down regulation of gene numbers between LVNC and LVTMEM176B. (**B**) The venn diagrams showed the overlap between LVNC and LVTMEM176B. (**C**) The upregulated GO-Cellular components between LVNC and LVTMEM176B were presented. (**D-E**) Cell adhesion molecules and the DNA replication signaling pathway were enriched as identified by GSEA analysis. The correlations between TMEM176B and cell adhesion molecules, as well as DNA replication in TCGA-OC (**F**) and RNA-seq (**G**) were examined using Pearson’s correlation analysis

To investigate the potential molecular mechanism of TMEM176B in OC, we analysed the RNA-seq gene expression profiles of A2780 cells overexpressing TMEM176B. Heatmap analysis revealed that TMEM176B overexpression resulted in a significantly different transcriptional profile. (Fig. S5). In addition, we identified 3,832 differentially expression genes (DEGs), including 1,855 genes with increased abundance and 1,977 genes with reduced abundance between LVTMEM176B group and LVNC group (Fig. 5A). The Venn diagram showed that 12,400 genes overlapped between the LVTMEM176B group and the LVNC group, with 519 genes specifically expressed in the TMEM176B overexpression group and 300 genes specifically expressed in the negative control group (Fig. 5B). Furthermore, upregulated GO-Cellular components indicated that cellular adhesion and junctions, cell morphology, and glucose metabolism were enriched in LVTMEM176B group (Fig. 5C). GSEA revealed enrichment in cell adhesion and DNA replication signaling pathways, with cell adhesion being upregulated and DNA replication being downregulated in the LVTMEM176B group (Fig. 5D-E). DNA replication was also enriched in the downregulated GO-Cellular component terms (Fig. S6).

Moreover, we performed a coexpression analysis in TCGA-OC and found that TMEM176B was strongly associated with genes related to cell adhesion and DNA replication, we selected the top 10 genes for mapping separately (Fig. 5F). RNA-seq data confirmed the above results. For example, the intercellular adhesion molecules such as ICAM1 and ARRB1, the vascular cell adhesion molecule VCAM1, immunoglobulin superfamily adhesion molecules such as CD4, and the integrin ITGAM were positively correlated with TMEM176B. However, replicator genes such as POLD2, RFC3, RFC5, MCM4, and MCM7 were negatively correlated with TMEM176B (Fig. 5G). Cell adhesion is the main factor affecting tumor metastasis [24]. When cell adhesion molecules lose control or mutate, a decrease in cell adhesion ability may occur, triggering metastasis [25]. Therefore, the positive regulation of cell adhesion by TMEM176B might be one of the reasons for its ability to inhibit OC cell metastasis.

### TMEM176B regulated the activation of the Wnt/β-catenin signaling pathway

**Fig. 6:**
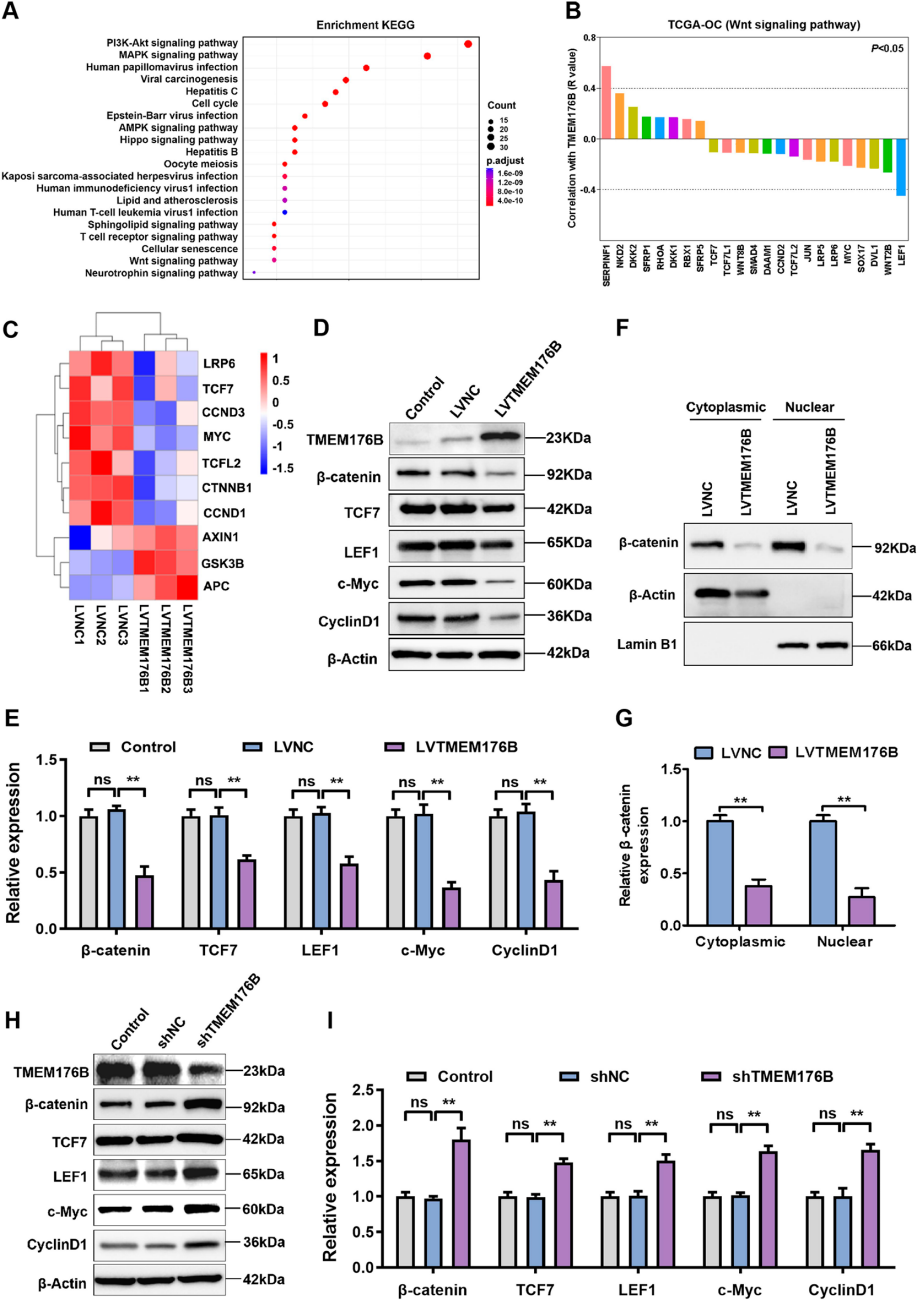
TMEM176B inhibited the activation of the Wnt/β-catenin signaling pathway. A2780 cells were transfected with negative control or TMEM176B overexpression lentiviruses. (**A**) The Wnt signaling pathway was enriched in KEGG pathway analysis. (**B**) The correlations between TMEM176B and Wnt signaling factors in TCGA-OC were examined using Pearson’s correlation analysis. (**C**) Heatmap of key genes in the Wnt signaling pathway between LVNC and LVTMEM176B. (**D-E**) Proteins expression of key genes in the Wnt signaling pathway were detected using Western blottingn in TMEM176B overexpression A2780 cells. (**F-G**) Protein expression of β-catenin in the cytoplasmic and nuclear were detected using Western blotting in TMEM176B overexpression A2780 cells. (**H-I**) Proteins expression of key genes in the Wnt signaling pathway were detected using Western blotting in TMEM176B knockdown OVCAR3 cells. **P < 0.01; ***P < 0.001, *n* = 3

KEGG analysis revealed that PI3K-Akt pathway and Wnt pathway were enriched in the TMEM176B overexpression group (Fig. 6A). Studies have shown that TMEM176B can regulate the growth of gastric cancer and triple-negative breast cancer by regulating the PI3K-Akt-mTOR pathway [15, 26]. As is well known, the Wnt pathway plays an important role in the occurrence and development of OC [27]. Specifically, upregulation of the Wnt pathway in OC has been shown to accelerate EMT-mediated metastasis [28]. However, the role of TMEM176B on OC by regulating Wnt signaling pathway has not been reported. CPTAC database analysis revealed a negative association between alterations in the Wnt pathway and TMEM176B in OC (Fig. S7).

To further characterize whether TMEM176B inhibited the progression of OC through regulating the Wnt signaling pathway, we performed a coexpression analysis of TMEM176B and Wnt signaling factors in TCGA-OC. We found that TMEM176B was positively correlated with Wnt pathway suppressor genes, such as SERPINE1 and NKD2, and negatively correlated with the Wnt pathway initiation genes LRP and DVL, the transcription factors TCF and LEF, and the downstream target genes c-Myc and Cyclin D1 (Fig. 6B). Our RNA-seq data also revealed that TMEM176B regulated the expression of key genes in the Wnt pathway (Fig. 6C). Next, western blotting analysis confirmed that TMEM176B overexpression significantly decreased the expression levels of β-catenin, TCF7, LEF1, c-Myc, and Cyclin D1, and inhibited the nuclear accumulation of β-catenin proteins in A2780 cells (Fig. 6D-G). Conversely, TMEM176B knockdown promoted the expression of Wnt signaling pathway-related proteins in OVCAR3 cells (Fig. 6H-I). Finally, via the protein interaction analysis database STRING, we identified interactions between β-catenin and proteins involved in cell adhesion and DNA replication (Fig. S8). These results suggest that TMEM176B suppresses the activation of the Wnt/β-catenin signaling pathway in OC.

### TMEM176B inhibited the proliferation and metastasis of OC cells through the Wnt/β-catenin signaling pathway

**Fig. 7:**
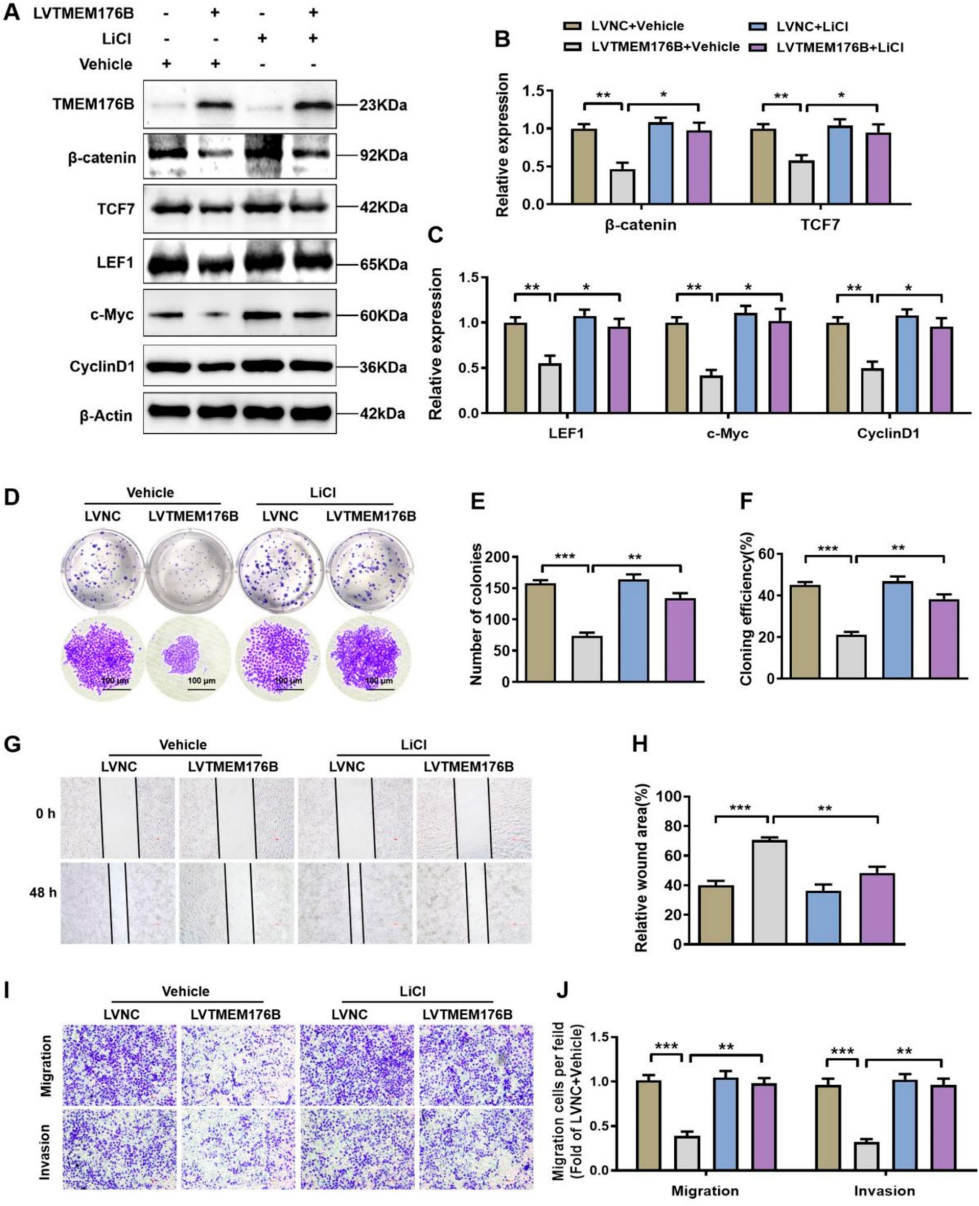
TMEM176B inhibited the proliferation and metastasis of OC cells via the Wnt/β-catenin signaling pathway. A2780 cells were transfected with negative control or TMEM176B overexpresion lentiviruses, and then incubated with LiCl (10 mmol/L) or vehicle (DMSO) for another 24 h. (**A-C**) Western blotting was used to determine the reversal efficiency of LiCl on the key proteins of the Wnt/β-catenin signaling pathway. (**D-F**) The impact of LiCl on the colony-forming ability. (**G-H**) The effect of LiCl on the migration was assessed by wound healing assays. The histogram showed the statistical analysis of the wound healing rate at 48 h. Scale bars, 500 μm. (**I-J**) The impact of LiCl on the metastasis was evaluated using transwell assay. Scale bars, 500 μm. *P < 0.05; **P < 0.01; ***P < 0.001, *n* = 3

Next, we utilized the Wnt/β-catenin pathway agonist LiCl to investigate whether TMEM176B inhibits the proliferation and metastasis of OC cells by negatively regulating the Wnt/β-catenin pathway. A2780 cells transfected with negative or TMEM176B overexpression lentivirus were treated with LiCl. Western blotting analysis revealed that the expression levels of β-catenin, TCF7, LEF1, c-Myc, and Cyclin D1 were decreased in the TMEM176B overexpression group, which was reversed by LiCl (Fig. 7A-C). Similarly, the colony formation assays demonstrated that LiCl restored the inhibition of cell proliferation induced by TMEM176B overexpression (Fig. 7D-F). Wound healing and transwell assays revealed that LiCl restored the inhibition of cell proliferation induced by TMEM176B overexpressionblocked the inhibitory effect of TMEM176B on cell migration and invasion (Fig. 7G-J). In summary, these findings indicate that TMEM176B inhibits proliferation and metastasis by suppressing the activation of the Wnt/β-catenin signaling pathway in OC.

### TMEM176B inhibited EMT through the Wnt/β-catenin signaling pathway

**Fig. 8:**
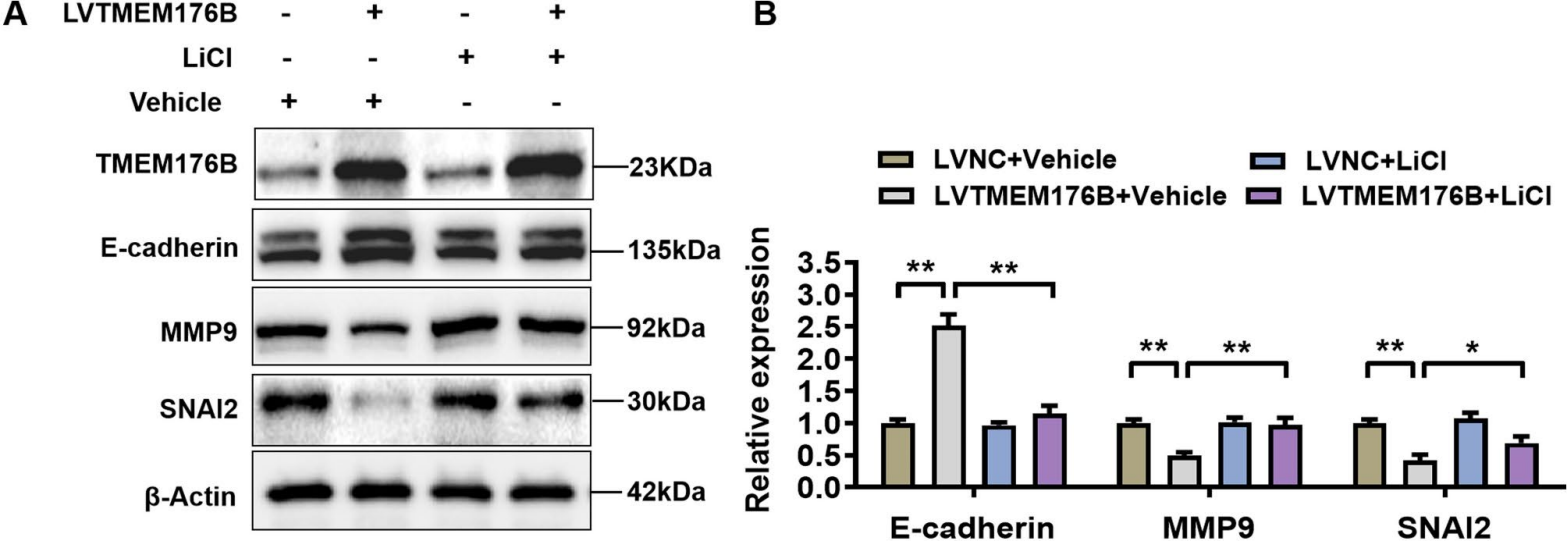
TMEM176B inhibits EMT via the Wnt/β-catenin signaling pathway. A2780 cells were transfected with negative control or TMEM176B overexpresion lentiviruses, and then incubated with LiCl (10 mmol/L) or vehicle (DMSO) for another 24 h. (**A-B**) Western blotting determined the reversal efficiency of LiCl on E-cadherin, MMP9, and SNAI2 in both the LVNC and LVTMEM176B groups. *P < 0.05; **P < 0.01, *n* = 3

EMT is a necessary process for tumor migration and progression [20]. We found that TMEM176B upregulated E-cadherin but downregulated MMP9 and SNAI2 in A2780 cells (Fig. 3K-L). A2780 cells overexpressing TMEM176B were subsequently treated with LiCl, and the expression of EMT markers were detected to evaluate whether TMEM176B inhibited EMT through the Wnt/β-catenin signaling pathway. The results showed that the inhibitory effect of TMEM176B overexpression on EMT was reversed by LiCl, resulting in decreased expression of E-cadherin and increased expression of MMP9 and SNAI2 (Fig. 8A-B). Thus, TMEM176B inhibited EMT through the Wnt/β-catenin signaling pathway in OC.

**Fig. 9:**
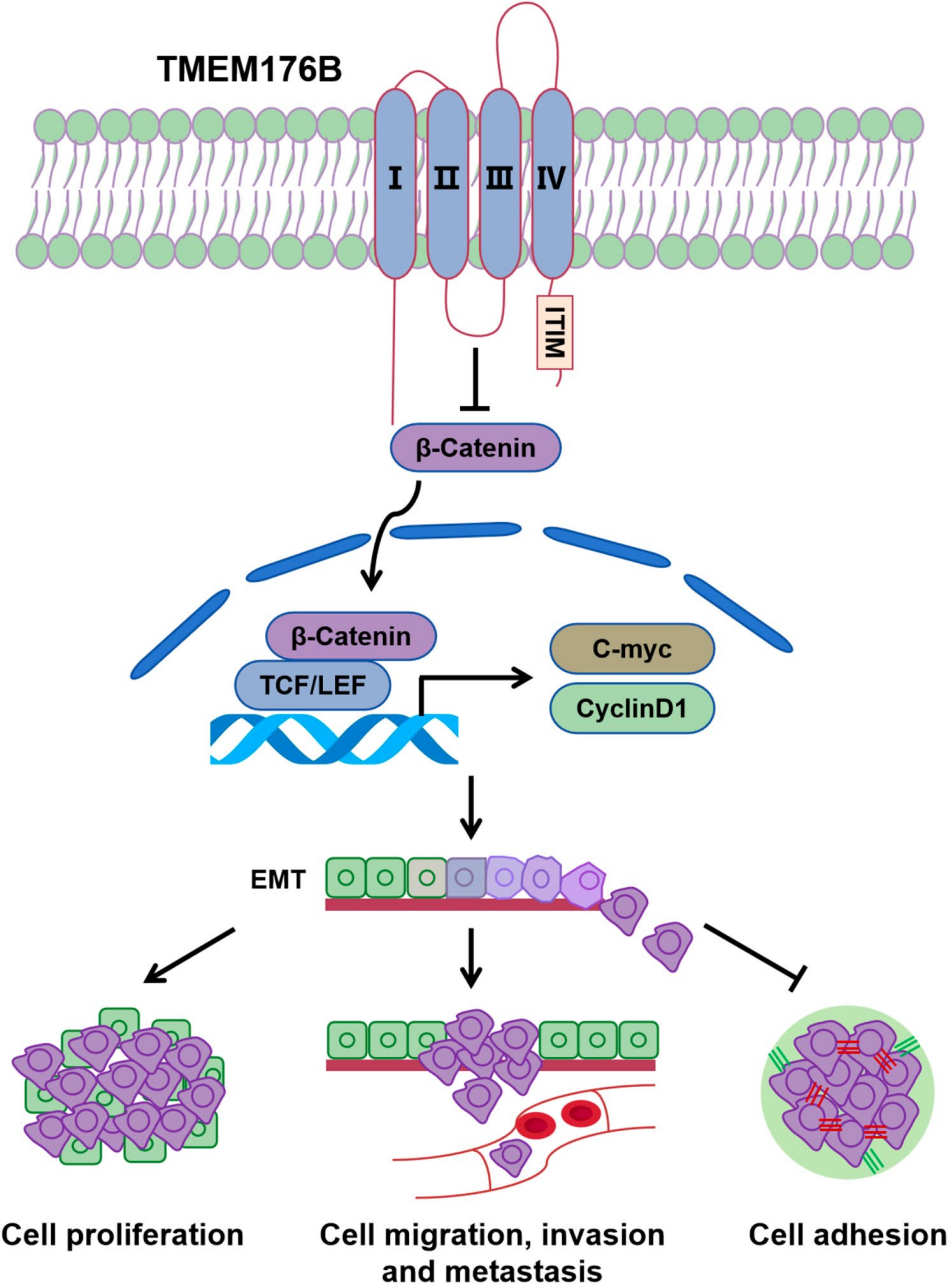
Schematic model illustrating the mechanism of TMEM176B inhibiting OC progression. In ovarian cancer, TMEM176B suppresses EMT by modulating the activation of the Wnt/β-catenin signaling pathway, thus significantly blocking OC progression

In our study, we demonstrated that TMEM176B suppressed EMT by modulating the activation of the Wnt/β-catenin signaling pathway, thus significantly blocking OC progression (Fig. 9).

## Discussion

Ovarian cancer (OC) is recognized as one of the deadliest gynecological tumors. About 75% of patients who are newly diagnosed with this condition present with either regional or distant spread of the disease, typically affecting other peritoneal structures such as the omentum, intestines, and peritoneal wall [29]. Despite advancements in various treatment strategies—including surgery, chemotherapy, radiation therapy, and immunotherapy—their effectiveness is often compromised by limited efficacy, side effects, and the emergence of drug resistance [30]. This situation underscores the pressing need to identify new therapeutic targets. Presently, there is a absence of research focusing on the role of TMEM176B in tumor cells [31]. In colorectal cancer, higher expression of TMEM176B is associated with lower overall survival rates [32]. Similarly, in breast cancer, TMEM176B promotes proliferation and migration by activating the PI3K/Akt signaling pathway [26]. Furthermore, Sun PH et al. found that found that TMEM176B is significantly overexpressed in lung adenocarcinoma and correlates with decreased overall survival outcomes. TMEM176B influences multiple cellular processes, such as cell proliferation, invasion, migration, and adhesion in vitro, as well as promoting tumor growth in vivo [22]. Collectively, these findings indicate that TMEM176B functions as a positive regulator in colon, breast, and lung adenocarcinomas. Conversely, Yang Y et al. found that TMEM176B acts as a negative regulator in prostate cancer, where its overproduction hampers the proliferation, invasion, and migration of LNCap cells [33]. These inconsistencies underscore the complex heterogeneity among different tumor types, highlighting the need for further investigation into the biology of different cancers. Based on these observations, our research sought to examine the role of TMEM176B in the progression of OC. In this analysis, we evaluated data from TCGA, and GTEx databases, finding a notable decrease in TMEM176B levels in OC, which is linked to adverse prognostic outcomes. Moreover, significant differences in TMEM176B protein expression were observed across various tumor stages (Fig. 1E), tumor grades (Fig. 1F), and patient age groups according to the CPTAC database. Thess findings suggest that TMEM176B serves as a vital diagnostic and prognostic marker. The low expression pattern of TMEM176B in OC was confirmed through the analysis of patient-derived ovarian tissue samples. Our research demonstrated through both in vivo and in vitro experiments that TMEM176B markedly hindered the progression of OC. We further established that TMEM176B has an essential role in OC, functioning as a negative regulator in the occurrence and progression of OC, and it may also serve as a potential target for therapy.

When EMT takes place, closely packed epithelial cells undergo transformation into loosely arranged mesenchymal cells, which leads to a decrease in intercellular adhesion and an improvement in migratory properties [34]. This enhanced metastatic potential in OC cells during the EMT phase is often associated with reduced intercellular adhesion, as well as the acquisition of migratory and invasive traits [18]. A significant decrease in E-cadherin expression is a critical marker of EMT [35]. In addition to E-cadherin, the activation of the snail family transcriptional repressor 2 (SNAI2) [36] and the heightened levels of matrix metalloproteinase 9 (MMP9) [37] are also vital elements involved in EMT throughout tumor progression. It has been observed that MMP9 exhibits high activity in advanced OC [38], and its increased expression plays a role in tumor cell migration, invasion, metastasis, and the development of ascites [39]. MMP9 directly cleaves E-cadherin [40], resulting in the loss of E-cadherin and the disruption of the integrity of intercellular connections, subsequently facilitating cell migration and invasion [41]. Our findings indicated that the knockdown of TMEM176B led to an decrease in E-cadherin levels with a increase in MMP9 and SNAI2 in OVCAR3 cells, while TMEM176B overexpression had the opposite effects, demonstrating that TMEM176B inhibited EMT in OC cells. The RNA-seq analysis highlighted that tubulin binding, actin binding, cell-matrix adhesion, cell-substrate junctions, and cell adhesion molecules were increased expression in the TMEM176B overexpression group. Additionally, the increase in E-cadherin expression further confirmed the enhancing effect of TMEM176B on cellular adhesion as observed in our experiments. These observations underscore the significance of TMEM176B in enhancing cell adhesion and promoting cell metastasis via EMT.

Several signaling pathways, including TGF-β, Notch, and Wnt/β-catenin [42, 43], contribute to EMT processes. The Wnt/β-catenin pathway is highly conserved through evolution, and its activation is recognized as a crucial modulator of EMT across various cancers [44], such as OC [45, 46]. Nonetheless, the precise molecular mechanisms by which Wnt signaling influences the progression and development of OC remain inadequately elucidated [47]. β-catenin serves as a principal regulator within the canonical Wnt signaling pathway; upon its accumulation in the cytoplasm, it translocates to the nucleus where it interacts with nuclear transcription factors TCF/LEF [48], thereby influencing cell adhesion, growth, and tumorigenesis [49]. Our findings indicated that the overexpression of TMEM176B inhibited the activity of the Wnt/β-catenin pathway in OC, leading to the degradation of β-catenin in the cytoplasm and blocking its translocation to the nucleus. Furthermore, the expression levels of transcription factors TCF7 and LEF1, along with downstream target genes such as c-Myc and Cyclin D1, were found to be reduced. RNA sequencing also demonstrated that TMEM176B overexpression led to increased mRNA levels of GSK3β, APC, and Axin1. The GSK3β/APC/Axin complex plays a vital role in managing the stability of β-catenin and is instrumental in its degradation [50]. Our experiments revealed that LiCl, a known activator of the Wnt signaling pathway, could restored the repressive effects of TMEM176B overexpression on the Wnt signaling pathway and mitigate the negative impacts on cell proliferation and metastasis. Concurrently, LiCl also reversed the suppression caused by TMEM176B overexpression on EMT.

## Conclusion

In summary, this research established the expression pattern of TMEM176B in OC and demonstrated its significant correlation with clinical factors. By controlling the activation of the Wnt/β-catenin signaling pathway, TMEM176B impeded EMT and consequently hindered the progression of OC. These results underscore the tumor-suppressive characteristics of TMEM176B in OC and emphasize its considerable promise for clinical utilization as a potential therapeutic option for this disease.

## Electronic supplementary material

Below is the link to the electronic supplementary material.

Supplementary Material 1

## Data Availability

The data that support the findings of this study are available from the corresponding author upon reasonable request.

## Abbreviations

OC: Ovarian cancer
TMEM176B: Transmembrane protein 176B
K‒M: Kaplan‒Meier
ROC: Receiver operating characteristic
AUC: Area Under Curve
RNA-seq: RNA sequencing
KEGG: Kyoto Encyclopedia of Genes and Genomes
GO: Gene Ontology
GSEA: Gene Set Enrichment Analysis
qRT: PCR-Quantitative real-time polymerase chain reaction
EMT: Epithelial-to-mesenchymal transition
TORlD: Tolerance-related and induced transcript
ITIM: Immunoreceptor tyrosine-based inhibitory motif
DEGs: Differentially expression genes
SNAI2: Snail family transcriptional repressor 2
MMP9: Matrix metalloproteases 9
