# Supplementary material for: TMEM176B inhibits ovarian cancer progression by regulating EMT via the Wnt/β-catenin signaling pathway
Source: J Transl Med. 2025 Mar 19;23:350. doi: 10.1186/s12967-025-06362-0 (PMC11921618; doi:10.1186/s12967-025-06362-0)
Supplement: Supplementary file 1 — Supplementary Material 1 [file 12967_2025_6362_MOESM1_ESM.docx]

**Supplementary data**

**TMEM176B inhibits ovarian cancer** **progression by regulating EMT via the Wnt/β-catenin signaling pathway**

Lili Yan^1,^ ^#^, Zhaona Song^1, #^, Lili Yi^1^, Conghui Tian^1^, Ruirui Zhang^3^, Xuying Qin^4^, Xiang Wang^5^, Shaoda Ren^6^, Xiaoping Ma^4^, Xiaobing Wang^4^, Xiaofeng Zhao^1^, Feifei Wang^1^, Jianmei Wei^1^, Xiaodong Jia^1^, Mingliang Gu^1^, Dianlong Jia^2, *^, Fengjiao Yuan^1, *^

*^1^ Joint Laboratory for Translational Medicine Research, Liaocheng People's Hospital, Liaocheng, Shandong 252000, PR China*

*^2^ State Key Laboratory of Macromolecular Drugs and Large-scale Preparation, School of Pharmaceutical Sciences and Food Engineering, Liaocheng University, Liaocheng, Shandong 252059, PR China*

*^3^ Department of Pathology, Liaocheng People's Hospital, Liaocheng, Shandong 252000, PR China*

*^4^ Department of Obstetrics and Gynecology, Liaocheng People's Hospital, Liaocheng, Shandong 252000, PR China*

*^5^ Central Laboratory of Liaocheng People's Hospital, Liaocheng, Shandong 252000, PR China*

*^6^ Shandong Provincial Key Medical and Health Discipline of Liaocheng Tumor Hospital, 252000, PR China*

*Corresponding authors

E-mail addresses: [121629304@163.com](mailto:121629304@163.com) (Fengjiao Yuan), jiadianlong@lcu.edu.cn (Dianlong Jia)

^#^ Contributed equally.

**
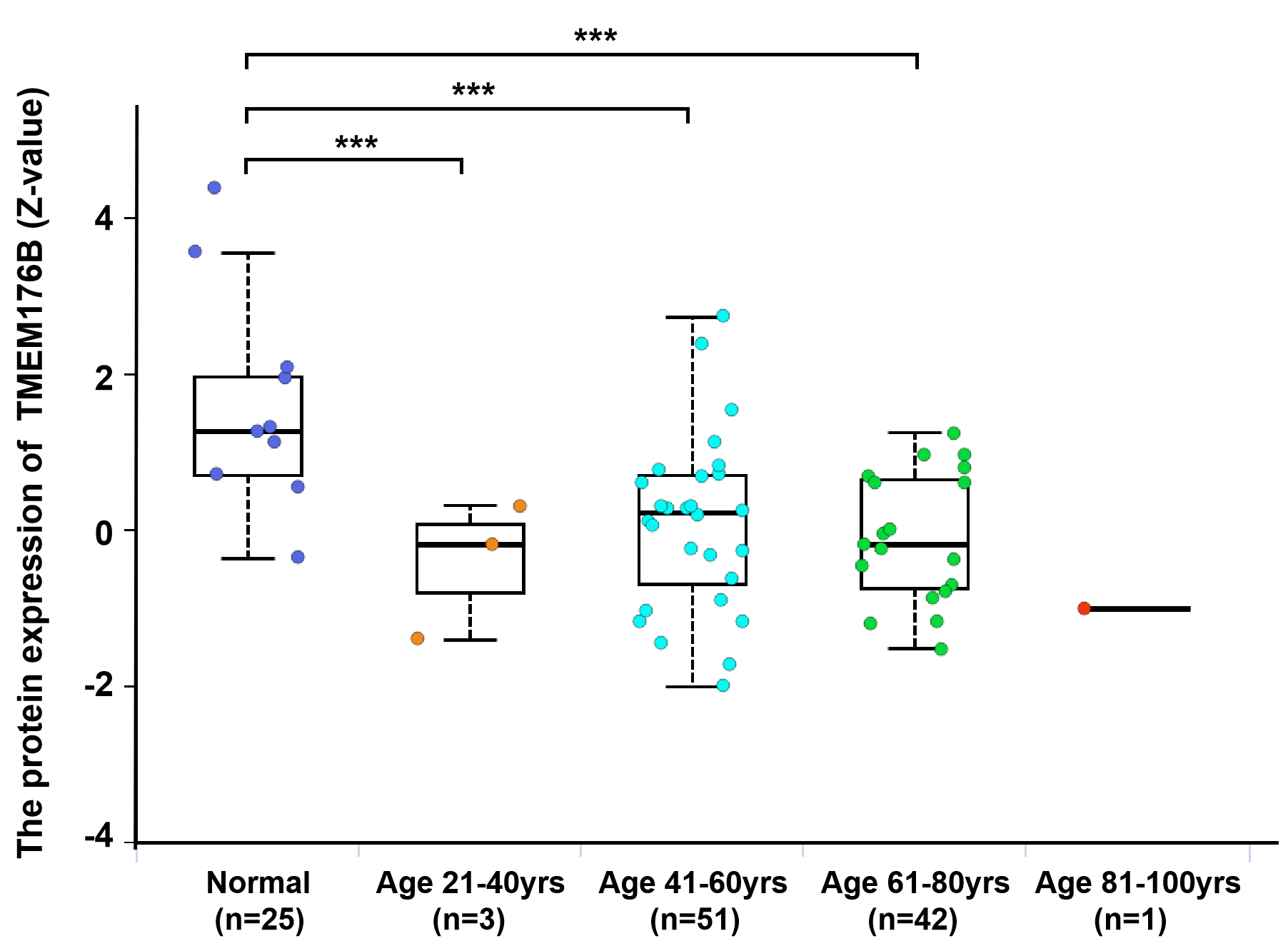
**

**Fig. S1** **Association between TMEM176B and patient age groups in OC.** The protein expression level of TMEM176B was significantly decreased in OC patients of different ages based on the CPTAC database. ****P* < 0.001.

**Fig. S2** The mRNA expression of TMEM176B in tumor tissue (Tumor; T) and contralateral normal ovarian tissue (Normal; N) from three OC patients. **P* < 0.05.


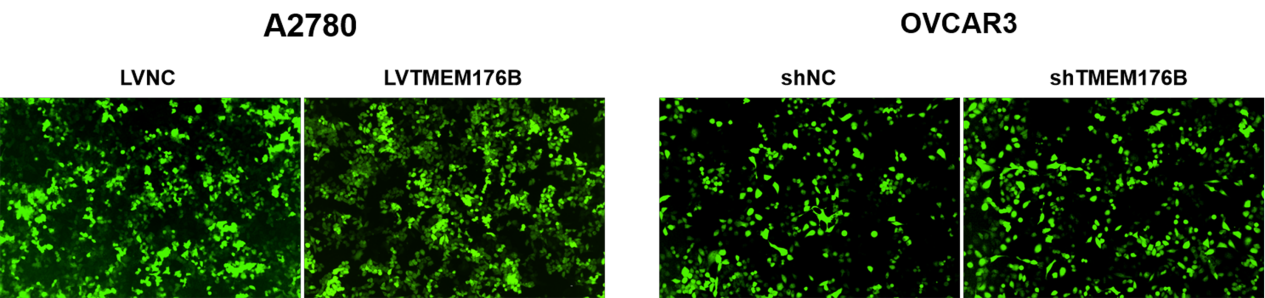


**Fig. S3 GFP fluorescence intensity in A2780 and OVCAR3 cells after lentivirus transfection.** Cells were transfected with negative control or TMEM176B lentiviruses. After 48 h of lentivirus transfection and selection with puromycin, the high levels of GFP fluorescence expression indicated that the plasmid has been successfully transfected into the cells. Scale bars, 500 μm.

**Fig. S4 Body weight changes in the xenograft nude mouse model.** During the monitoring of tumor growth, the body weight of mice in both groups was not adversely affected.


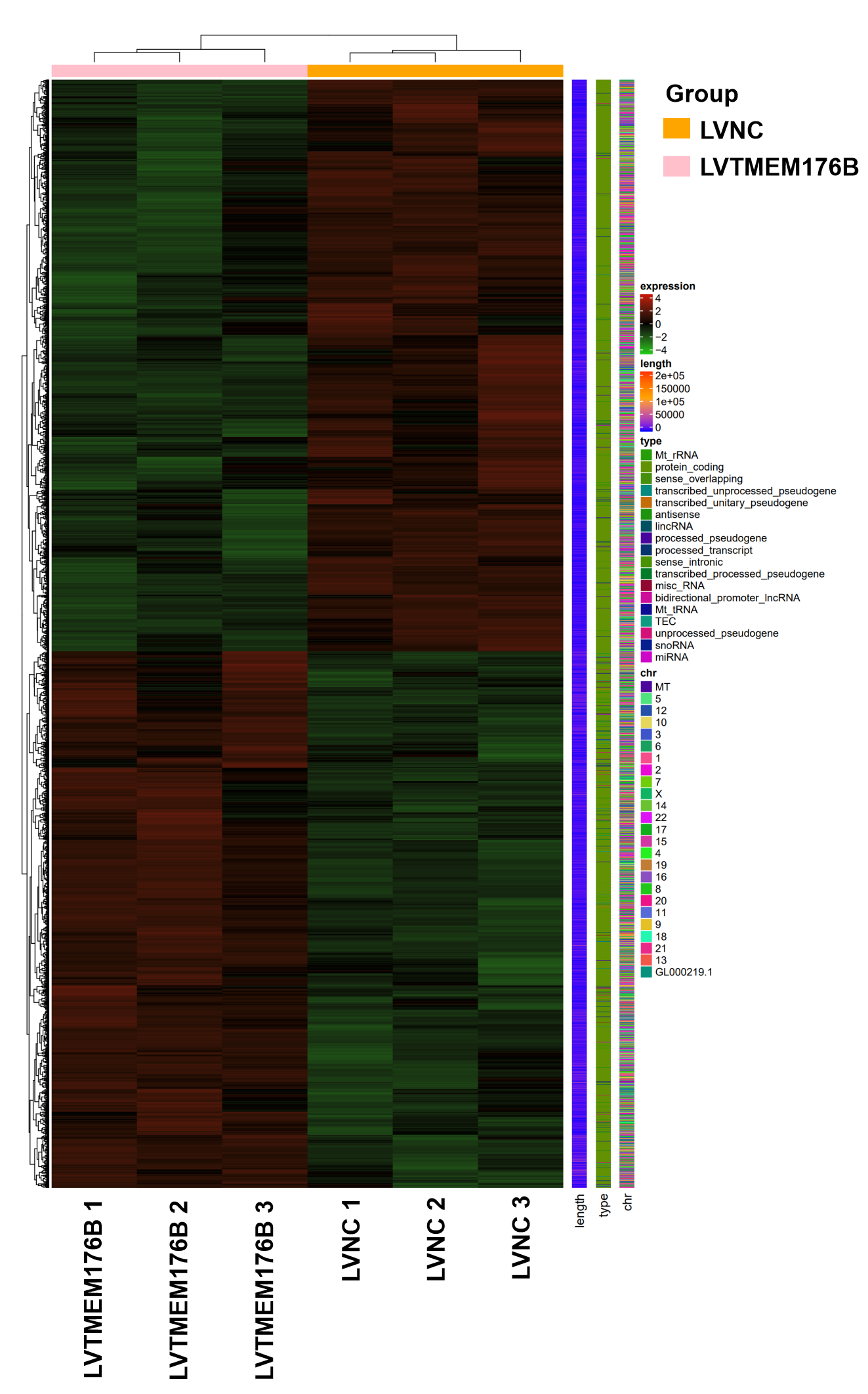


**Fig. S5 Heatmap of the LVNC group and LVTMEM176B group.** RNA-seq analysis revealed completely different transcriptomes in the A2780 cells with TMEM176B overexpression.


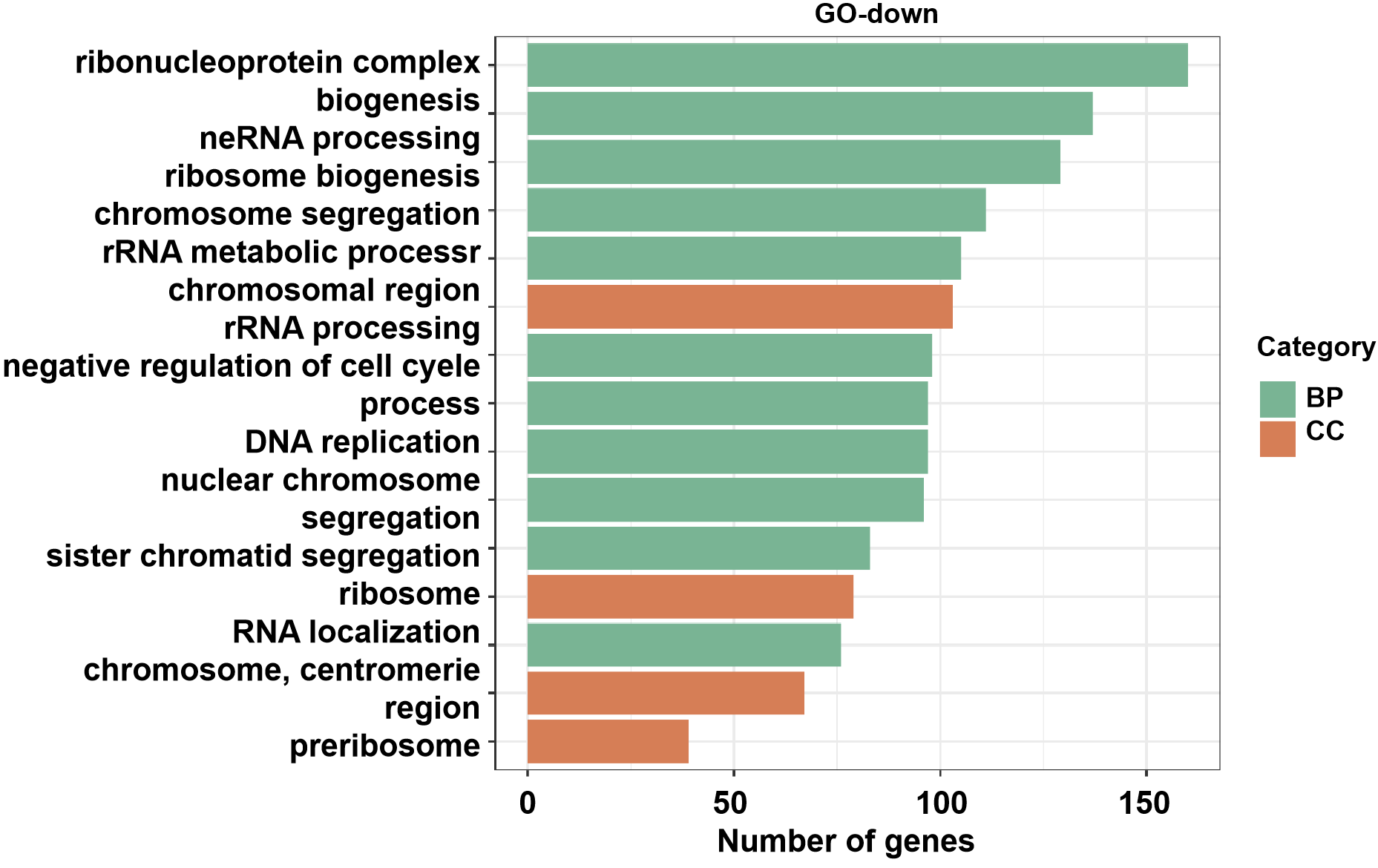


**Fig. S6 Down-regulated GO-Cellular components in the LVTMEM176B group.** RNA-seq analysis revealed that DNA replication was found enriched among the downregulated GO-Cellular components


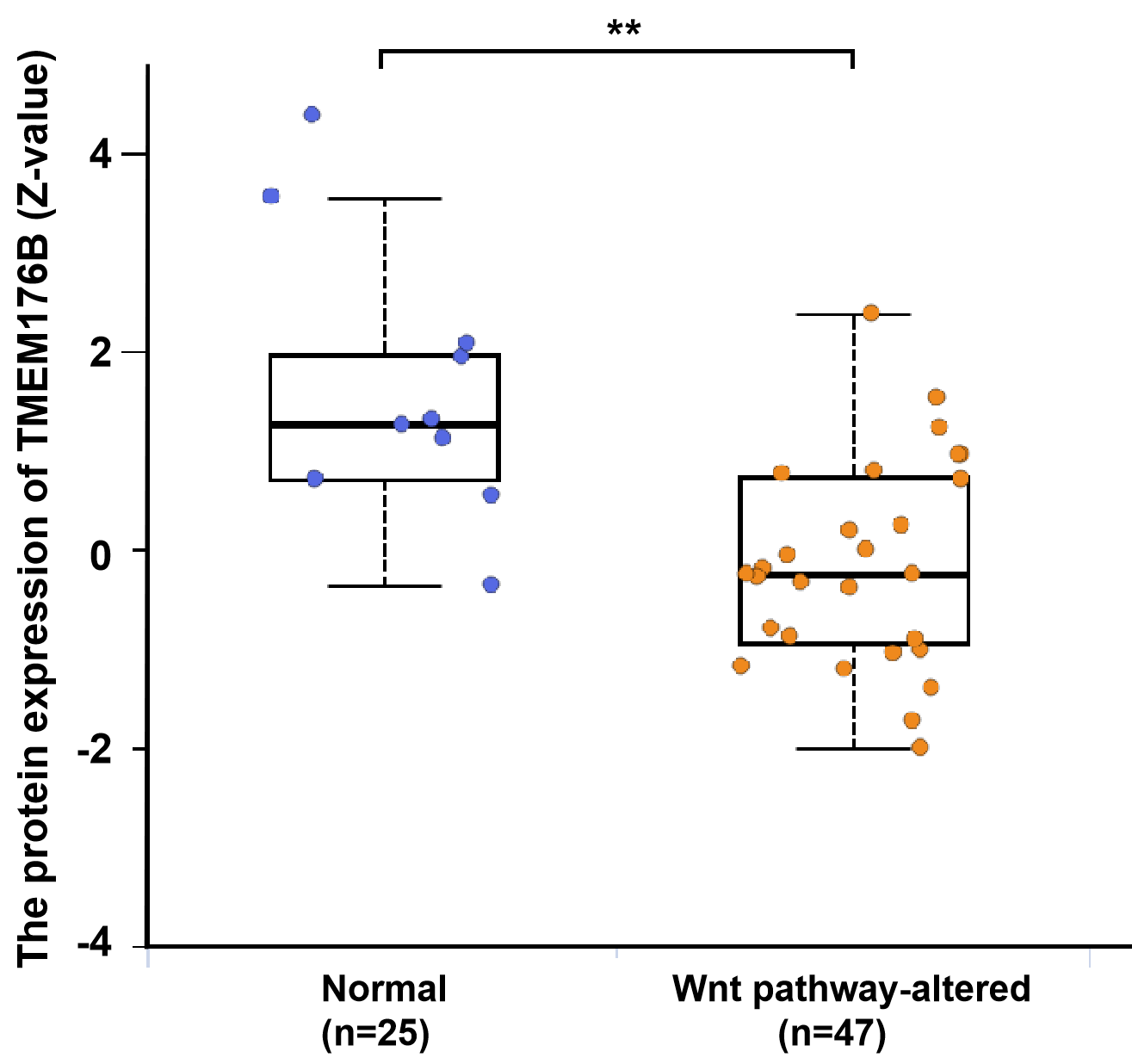


**Fig. S7 Association between TMEM176B and Wnt pathway in OC.** Alterations in the Wnt pathway in OC affect the protein level of TMEM176B based on the CPTAC database. ***P* < 0.01.


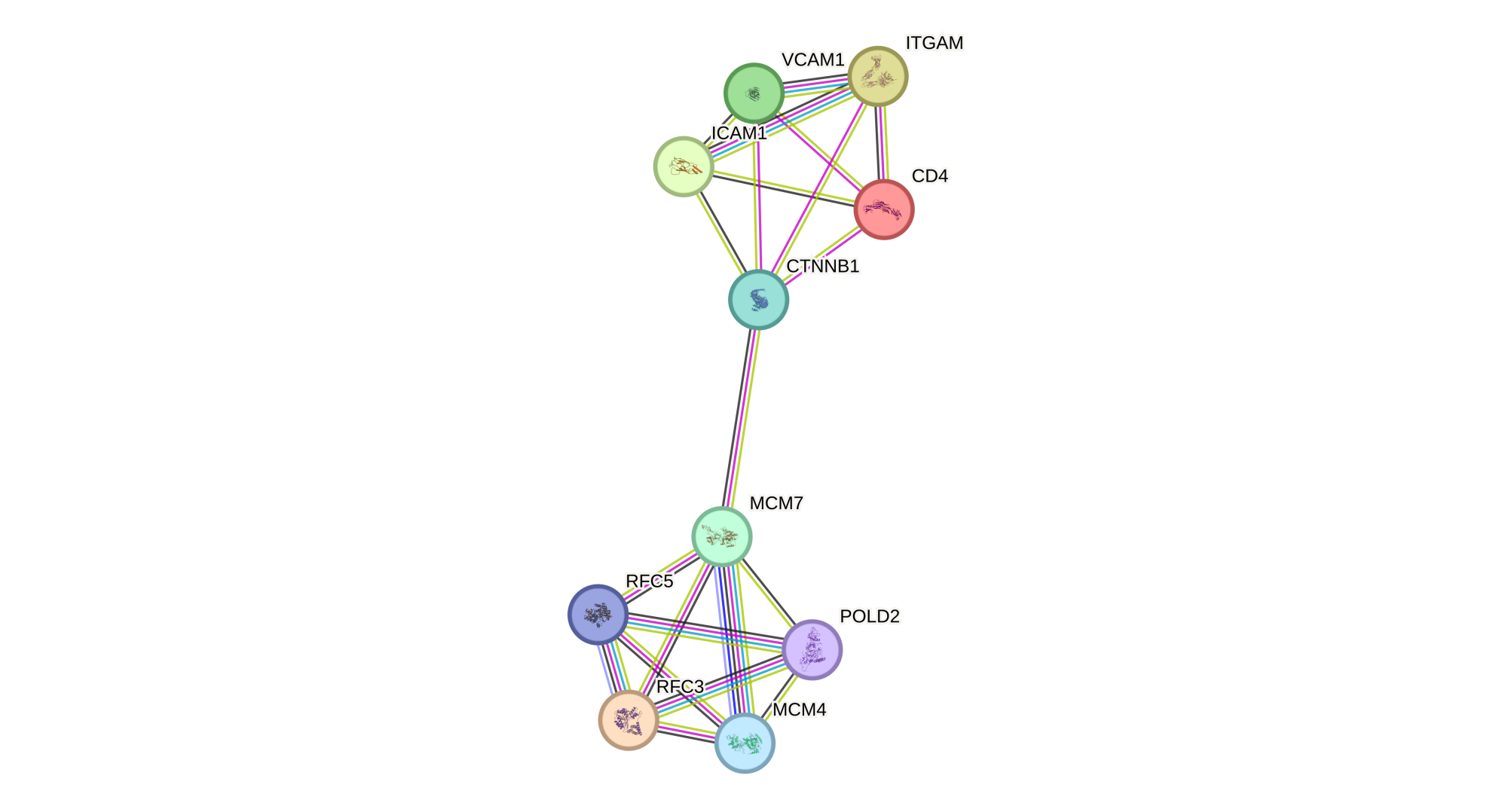


**Fig. S8** **PPI network was constructed by STRING database.** The interaction between β-catenin and key proteins involved in cell adhesion and DNA replication were analysed.
